# Can introducing a direct endocrine pathway reduce hyponatraemia in elective knee and hip replacements? A closed-loop audit and service evaluation study

**DOI:** 10.1308/rcsann.2021.0296

**Published:** 2022-02-17

**Authors:** M Waller, S Barkley, T Harrison

**Affiliations:** Sheffield Teaching Hospitals NHS Foundation Trust, UK

**Keywords:** Hyponatraemia, Total hip replacement, Total knee replacemen, Endocrinology pathway, Service evaluation

## Abstract

**Introduction:**

Hyponatraemia has a prevalence of up to 30% after orthopaedic surgery and is associated with poor outcomes, including around 20% mortality and longer hospital stays. This study assessed the prevalence of hyponatraemia following total hip and knee replacement, the causes, further tests, management, effect on length of stay, intensive care admissions and the impact of an endocrinology hyponatraemia protocol.

**Materials and methods:**

Day one postoperative urea and electrolyte results for patients undergoing elective total hip and knee replacements were reviewed. Retrospective data was gathered through the web-based requesting and reporting system ICE. Parameters included demographics, procedure, sodium pre- and postoperatively, endocrine input, high-dependency admissions and length of hospital stay. Next, a hyponatraemia protocol based on NICE guidance was developed with the endocrinology department and a second audit cycle was initiated. SPSS software was used to analyse the data.

**Results:**

Hyponatraemia occurred in 12% of patients, resulted in a significantly longer stay (7.7 days vs 4.6, *t* –4.6, *p* < 0.00001) and multiple critical care admissions (8 days). It was more common in total knee replacement (chi square 5.5194, *p* = 0.018807) and older age (*t* –2.81083, *p* = 0.002619). Prior to implementation of the endocrine pathway, hyponatraemia was under-investigated (38%). The precipitating factors such as age and use of diuretics corroborated with prior research. Implementation of the hyponatraemia protocol resulted in quicker endocrinology referrals (2.3 vs 3.6 days), reduced length of stay by 0.7 days (*t* –2.40973, *p* = 0.008144) and reduced intensive care days to 0 (chi square 4.6189, *p* = 0.031622).

**Discussion:**

This study found a similar incidence of hyponatremia as earlier research with the same precipitating factors, the only exception being an increased incidence in patients undergoing knee compared with hip replacemenr The introduction of the direct endocrine pathway proved to be safe and effective without increasing local workload significantly. The main limitation in this project was the fact that it was carried out in a single unit, although this process could be easily replicated should other units wish to adopt it and compare results over a wider cohort.

**Conclusions:**

This endocrine pathway is easily reproducible for other departments. It may help reduce waiting times and improve outcomes for total hip and knee replacements within the NHS.

## Introduction

Hyponatraemia is a common problem in geriatric patients attending hospital,^1^ and although it is most often a complication of medical pathology such as cardiac failure, renal failure, liver failure and pneumonia,^2^ it has a high prevalence of up to 30% following elective orthopaedic procedures.^3,4^ Hyponatraemia is associated with poor outcomes postoperatively,^5^ and is associated with increased mortality even when mild but especially with cases of profound hyponatraemia.^6^ When conducting a literature search on hyponatraemia in elective arthroplasty it was found that there have been a number of papers highlighting the negative impact of this condition on outcomes and the need for prompt recognition and management to reduce this impact, but no papers looking at tools to assist in the recognition and management of hyponatraemia and the impact this could have on length of hospital stay and need for intensive care input.

The aetiology of hyponatraemia following surgery in the elderly is complex and multifactorial, but proposed mechanisms include syndrome of inappropriate antidiuretic hormone secretion, hypervolaemia, hypovolaemia and drug related factors.^4^ Factors that significantly increase the risk of developing postoperative hyponatraemia in orthopaedic surgery are increased age, hip arthroplasty, use of thiazide diuretics and the amount of lactated Ringer’s solution (Hartmann’s in the UK) infused.^7,8^ The prevalence of hyponatraemia in arthroplasty patients was found to be higher than in other surgical inpatients in a Belfast study,^8^ but there are limited data for departments in England.

The initial study’s objective was to ascertain the prevalence and causes of postoperative hyponatraemia in patients undergoing elective lower limb arthroplasty and the impact this has on intensive care admissions and length of hospital stay. The second loop of the cycle aimed to evaluate the impact that implementing a specific endocrinology pathway to manage hyponatraemia had on length of stay and intensive care admissions. We hypothesised that improving the efficiency of managing hyponatraemia by streamlining the referral process to endocrine and subsequent management would reduce the length of stay of these patients and reduce the need for intensive care input.

## Materials and methods

The European Society of Endocrinology developed the joint European guideline for the management hyponatraemia in conjunction with the European Society of Intensive Care Medicine and the European Renal Association-European Dialysis and Transplant Association.^2^ This guideline splits hyponatraemia into three categories: mild (130–135mmol/l as measured by ion-specific electrode), moderate (125–129mmol/l) and profound (<125mmol/l).^2^ For the purpose of this study, a serum sodium level of 133mmol/l was chosen as a cut-off point, following to our local guidance.

The postoperative renal function results for all patients who attended for elective primary total hip or knee replacements (THR and TKR) on one unit within Sheffield Teaching Hospitals NHS Foundation Trust between May and July 2018 were reviewed. Retrospective data were gathered by the main author (MW) online via the laboratory results recording system and patient records. Parameters included patient demographics (age and sex), type of arthroplasty performed and sodium levels on admission, day one and day three postoperatively and discharge, an osmolality assessment if an endocrine referral was made during admission, information on critical care admissions and length of hospital stay. Every patient attending the unit for arthroplasty during the above period was included in the study; patients attending the unit but not having arthroplasty were excluded from the study.

Following the results of the initial audit, a new hyponatraemia protocol based on the National Institute for Health and Care Excellence (NICE) guidance was developed by the main author in association with the endocrinology department and a second audit cycle was initiated from October to February 2018 using the same methods as the first cycle. The second cycle results were collected and analysed after all patients attending during this period had been discharged from the unit. No further patient follow-up was carried out.

All patient data were anonymised using hospital identification only and data was stored on the hospital’s secure server in a Microsoft excel spreadsheet. Statistical analysis was performed using SPSS software. Statistical significance was assumed at *p* < 0.05 for the two tests used (*t*-test and chi square test). The project was registered with and approved by the local clinical effectiveness unit at Sheffield Teaching Hospitals Trust, reference number 8818.

## Results

The first audit cycle included 295 patients. Their average age was 69 years (range 31–93 years) and 62% of patients were women (183). The average length of stay in hospital was 4.6 days (range 1–25 days). A total of 168 (57%) patients had a TKR and 127 (43%) had a THR.

In total, 36 (12%) patients developed hyponatraemia. Of these, 15 developed mild, 10 moderate and 11 profound hyponatraemia. Some 82% of cases of hyponatraemia were discovered before day 2 postoperatively, although on average their sodium was then not rechecked until after day 4 of their admission. It took an average of 3.6 days (range 1–9 days) for a referral to the endocrine department. Thirty-eight percent of patients with hyponatraemia had it investigated; all of them had a cause identified. Thirty three percent of patients with hyponatraemia had endocrine input.

The average length of stay for all patients who developed hyponatraemia (mild, moderate or severe) was significantly longer than those who did not at 7.7 (1–25) days versus 4.6 (1–25) days (*t*=–4.6 *p*<0.00001).

Patients undergoing TKR were more likely to develop hyponatraemia (mild, moderate or severe) than those having a THR (28 vs 8, chi square 5.5194, *p* = 0.019). Women were more likely to develop hyponatraemia than men (26 vs 10), but the result was not statistically significant (chi square 1.5227, *p* = 0.217209). Older patients were statistically more likely to develop hyponatraemia; the average age was 74.5 years (range 56–86 years) compared with 69.1 years (range 31–93 years) for those who did not develop hyponatraemia (*t* –2.81083, *p* = 0.002619).

**Figure 1:**
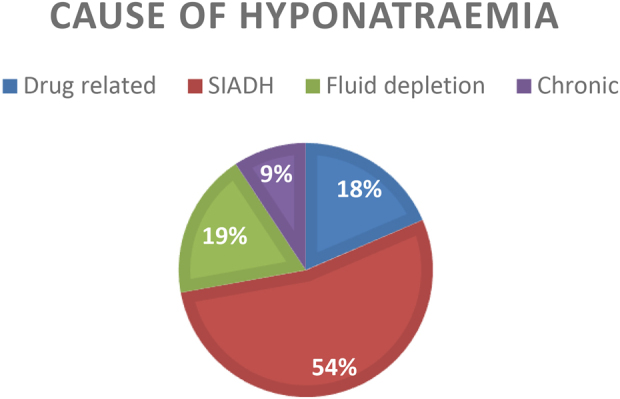
Causes of profound hyponatraemia in the first cycle as percentages

**Figure 2:**
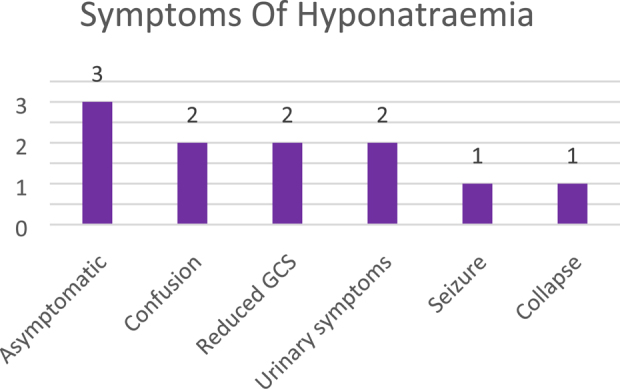
Symptoms exhibited by profoundly hyponatraemic patients. GCS = Glasgow Coma Scale.

Of the 11 profoundly hyponatraemic patients (Na < 125 mmol/l), 4 were admitted to critical care with a total combined stay of 8 days. Profound hyponatraemia was found to be more common in patients undergoing TKR (8 patients, 72%) and in women (8 patients 72%). Ten (90.9%) patients with profound hyponatraemia had investigations for the cause, with 9 electronically referred to endocrinology. Eight of the 11 profoundly hyponatraemic patients remained hyponatraemic on discharge (although they had improved) and the average length of stay for this group was 10.7 days (range 4–25 days). The causes of hyponatraemia and clinical features in profoundly hyponatraemic patients are shown in Figures 1 and 2. No patients died due to hyponatraemia during the first audit cycle.

**Figure 3:**
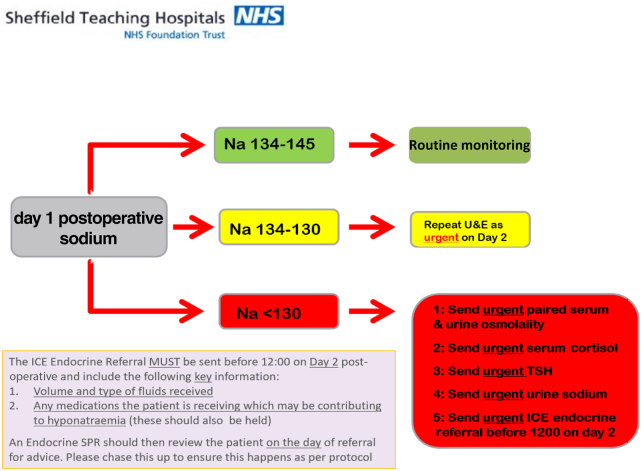
Hyponatraemia protocol used on the elective arthroplasty ward for the duration of the second audit cycle. Na = sodium; SPR = specialist registrar; TSH = thyroid-stimulating hormone.

Following the results of the first cycle, a dedicated endocrine pathway was produced, aiming to reduce the length of stay and critical care admissions of patients with hyponatraemia. This pathway included the poster below which was displayed in the clinical areas and an agreement with endocrine to see arthroplasty patients referred to them via the web-based requesting and reporting system ICE on the same day (Figure 3).

The second audit cycle included 263 patients. The average age of patients was 68 years (range 20–89 years) with an average length of stay of 3.9 days (range 1–25 days); 155 (59%) patients had a TKR and the percentage of male versus female patients was comparable to the first cycle.

In the second cycle, 31 (11.7%) patients developed hyponatraemia. Of these, 12 developed mild, 16 moderate and 3 profound hyponatraemia. All patients who were profoundly hyponatraemic had their hyponatraemia investigated, a referral to endocrinology and a cause identified.

As in the first cycle, hyponatraemia was significantly more common in TKR compared with THR (24 patients vs 7, chi square 4.2515, *p* = 0.039216) and was more common in older patients (*t* 3.09282, *p* = 0.001087).

All three of the patients who were profoundly hyponatraemic (serum sodium of <125mmol/l) underwent TKR, two were female and none admitted to critical care. All had their hyponatraemia diagnosed before postoperative day 2 and all had their sodium rechecked on day 3. It took an average of 2.3 days (range 1–5 days) for a referral to endocrine (1.3 days less than in the first cycle). The average length of inpatient stay for patients who were profoundly hyponatraemic was just 5.3 days (2–25 days), less than half that in the first cycle.

**Figure 4:**
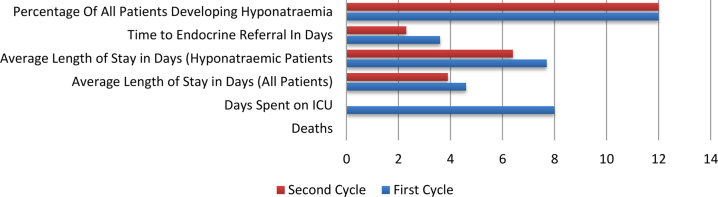
Comparison of first and second cycle results. ICU = intensive care unit.

The causes of hyponatraemia identified were syndrome of inappropriate antidiuretic hormone secretion, hypovolaemia, hypervolemia, medication related and chronic. Symptoms exhibited and fluids given were not assessed in the second cycle. There was no follow-up for patients following completion of both cycles. A comparison of the results of the two cycles is shown in Figure 4.

## Discussion

This service improvement project demonstrates that introducing a direct endocrine referral pathway to be used in patients undergoing elective hip and knee replacements can be safe and effective. The average length of stay for all patients undergoing hip and knee arthroplasty who developed hyponatraemia was significantly lower in the second cycle following the introduction of the endocrine pathway.

During both the first and second cycles, hyponatraemia was found to be common following elective arthroplasty surgery, with 12% of patients demonstrating some degree of hyponatraemia on their postoperative blood results. Eleven percent of hyponatraemic patients (4/36) became profoundly hyponatraemic requiring admission to intensive care in the first cycle but, by following the simple endocrine referral pathway used in the audit, we were able to prevent the need for any admissions to intensive care during the second cycle.

Hyponatraemia was found to be consistently more common in patients who underwent TKR rather than THR in both cycles, which differed from previous research,^3,8^ although the cause for this difference is unclear and was not investigated in this study. In both cycles, the precipitating factors identified (such as age and use of diuretics) were similar to those identified in previous research.^7–9^ A study by Greco *et al* suggested that postoperative bloods are only required in patients with low preoperative measured levels and in patients with medical comorbidities.^10^ However, this study only focused on postoperative haemoglobin and potassium levels. Following this audit, we believe it is important to also consider both the type of surgery, age of patient and medications the patient is receiving to be additional risk factors when specifically investigating low sodium.

The reason why the endocrine pathway was so effective is probably multifactorial. By using a dedicated endocrine referral pathway, all patients receive standardised investigations and treatments based on the cause of the hyponatraemia. It allows for earlier investigations and specialist review, which probably results in early correction of the low sodium, through both fluid management and preventing the use of potentially detrimental medications. The pathway used is reproducible and could be implemented at other units dealing with elective arthroplasty patients. There were no previous studies identified looking at the effect of earlier specialist endocrine input on hyponatraemia outcomes for comparison.

This main limitation of this study is that it was based solely within one unit limiting its relevance to the wider population without further research. This study only assessed patients over four-month periods limiting the reliability of the data which could be improved by assessing patients over a wider timescale.

## Conclusion

The use of a dedicated endocrine pathway for the management of postoperative hyponatraemia can significantly reduce length of stay, intensive care admissions and morbidity in elective arthroplasty patients. Given the significant improvements we observed in patient care, we advise all units managing postoperative elective arthroplasty consider adopting a similar pathway.
